# Awareness and implementation of nine World Health Organization’s patient safety solutions among three groups of healthcare workers in Oman

**DOI:** 10.1186/s12913-016-1771-1

**Published:** 2016-09-30

**Authors:** Ahmed Al-Mandhari, Ibrahim Al-Zakwani, Samir Al-Adawi, Samra Al-Barwani, Lakshmanan Jeyaseelan

**Affiliations:** 1 Department of Family Medicine & Public Health, Sultan Qaboos University Hospital, Sultan Qaboos University, Muscat, Sultanate of Oman; 2 Department of Pharmacology & Clinical Pharmacy, College of Medicine & Health Sciences, Sultan Qaboos University, Muscat, Sultanate of Oman; 3 Department of Behavioral Sciences, College of Medicine and Health Sciences, Sultan Qaboos University, Muscat, Sultanate of Oman; 4 Directorate General of Quality Assurance Center, Ministry of Health, Muscat, Sultanate of Oman; 5 Department of Statistics and Health Information, Lakshmanan Jeyaseelan, Sultan Qaboos University Hospital, Muscat, Sultanate of Oman

**Keywords:** WHO, Patient safety solutions, Patient safety, Knowledge, Attitude and practice, Nine life-saving patient safety solutions

## Abstract

**Background:**

The pressing need to reduce burgeoning poor safety measures affecting millions worldwide has alerted World Health Assembly to set-up mechanisms to increase patient safety. In response to such needs, World Health Organization (WHO) formulated nine life-saving patient safety solutions that would be essential to lower reduce healthcare-related harm. There is a paucity of research examining awareness of such nine patient safety solutions. This study has been designed and conducted to compare self-estimated awareness and practice of the World Health Organization’s nine “Life-saving Patient Safety Solutions” aide memoirs among different groups of healthcare workers in Oman.

**Methods:**

All nationwide healthcare workers (nurses, physicians and allied health professionals) in hospitals and primary healthcare under the auspice of Ministry of Health were the target population of this survey. Participants were selected by a simple, systematic random sampling from the list of staff in each representative institution. The study was conducted from November 2012 to February 2013. A total of 800 participants (590 from health centers and 210 from hospitals) were invited to participate in this study.

**Results:**

A total number of 763 healthcare professionals consented to participate. The overall response rate was 95 % with the majority being nurses, female staff and who had﻿ an average of more than 4 years of experience. Overall, 85 % of the participants self-estimated awareness of the nine life-saving patient safety solutions showed the nurses being the most aware, followed by physicians with the allied health professionals showing suboptimal awareness. The primary healthcare center staff demonstrated higher awareness compared to hospital staff. There was a complex relationship between health professional’s age, place of work and awareness and practice.

**Conclusion:**

This study lays the foundation for international comparisons of self-estimated awareness and practice towards nine patient safety solutions. The data from Oman indicates the need for more attention to be directed towards heightening awareness and practice of the nine patient safety solutions.

**Electronic supplementary material:**

The online version of this article (doi:10.1186/s12913-016-1771-1) contains supplementary material, which is available to authorized users.

## Background

Despite distinctive improvements in healthcare delivery in emerging economies, recent reports suggest that the progress might be hindered by existing sub-optimal safety measures essential for best practice in delivering healthcare [1]. In response to this trend, the World Health Assembly ‘Resolution 55.18’ in 2004 proposed to set-up mechanisms to increase what was termed ‘life-saving patient safety solutions’ [2]. The World Health Organization (WHO) formulated nine life-saving Patient Safety Solutions Aide memoirs in order to implement actions that address risks associated with particular patient safety problems and reduce healthcare-related harm, affecting millions of patients worldwide [3, 4]. The nine life-saving Patient Safety Solutions Aide memoires aim to address errors or adverse events related to inappropriate catheterization, poor cooperative behavior and communication among healthcare providers, healthcare-associated infection equipment failure, unsafe injection devices, medication errors, failures in patient identification systems and patient transfer, concentrated use of electrolyte solutions and wrong site surgery [5–8]. While the importance of these safety measures have been widely acknowledged [9], there is a dearth of information on how life-saving patient safety solutions are perceived by existing health practitioners. Therefore, it would be of paramount importance to raise the awareness of nine life-saving Patient Safety Solutions among health care workers.

The study aims to compare the self-estimated awareness and practice of the nine Patient Safety Solutions Aide memoires and hospitals and health centers in Oman (a country located in the southeastern coast of the Arabian Peninsula with a population of approximately 2.23 million Omani nationals and 1.76 expatriates [10]). The classification of health care workers specifically entail nurses, physicians and allied health professionals such as pharmacists, physical/occupational/speech therapists, biomedical scientists, and dieticians. The interrelated aim of this study is to raise awareness and ensure the proper implementation of the nine life-saving patient safety solutions.

## Methods

### Study design

The survey is cross-sectional and designed to assess the knowledge, attitude and practice of the nine patient safety solutions among three cadres of health workers in Oman (nurses, physicians, and allied health professionals). It was conducted during the period of November 2012 to February 2013. In order to ensure an adequate response rate, the survey questionnaires were first sent to the Directors General and Hospital Executive Directors. The survey questionnaires were subsequently sent to a focal point, the National Patient Safety project. They facilitated the distribution, collection, and submission of the survey feedback to the Department of Quality Assurance & Patient Safety, Ministry of Health (MoH).

### Assessment tool

A questionnaire was developed to tap into self-estimated awareness and practice of nine life-saving patient safety solutions. The developed questionnaire is composed of three sections. The content of the questionnaire was theoretically informed by a literature review exploring patient safety solutions as previously expounded by the WHO [7]. During the construction of the questionnaire, nine patient safety solutions were reformulated as questions into a three point Likert-type instrument, featuring ‘Agree’, ‘Disagree’ or “I do not know”. Nine items of the patient safety solution were worded to capture self-estimated awareness and a similar number were worded to capture practice as shown in Table 1 and Additional file 1. For simplicity, the responses were clustered into ‘Yes’ or ‘No’.

**Table 1:** Demography and level of self-estimated awareness among different subtypes of healthcare workers in Oman (*N* = 763)

Characteristics	Nurses (*n* = 351)	Physicians (*n* = 180)	Allied health professionals ^a^(*n* = 232)	*P* value
Demographic				
1. Age, mean ± SD, years	32 ± 7	39 ± 10	32 ± 7	<0.001
2. Female gender, n (%)	315 (90 %)	55 (31 %)	130 (56 %)	<0.001
3. Numbers of years at this hospital median (IQR), years	5 (2–10)	3 (1–7)	5 (2–10)	<0.001
4. Numbers of years at this hospital in the present specialization/unit, median (IQR), years	3 (1–7)	3 (1–5)	4 (1–8)	0.065
Awareness				
1. Have you heard about patient safety solutions before?	321 (91 %)	150 (83 %)	187 (81 %)	<0.001
a. Patient identification	279 (79 %)	125 (69 %)	153 (66 %)	0.001
b. Look-alike sound-alike medication names (LASA)?	158 (45 %)	73 (41 %)	80 (34 %)	0.040
c. Improved hand hygiene to prevent health-care associated infections (HCAI)?	293 (83 %)	141 (78 %)	143 (62 %)	<0.001
d. Performance of correct procedure at correct body site	228 (65 %)	104 (58 %)	83 (36 %)	<0.001
e. Avoiding catheter and tubing mis-connections	147 (42 %)	82 (46 %)	48 (21 %)	<0.001
f. Control of concentrated electrolyte solutions	119 (34 %)	69 (38 %)	48 (21 %)	<0.001
g. Communication during patient handovers	237 (68 %)	108 (60 %)	111 (48 %)	<0.001
h. Assuring medication accuracy at transitions in care	212 (60 %)	88 (49 %)	71 (31 %)	<0.001
i. Single use of injection devices	262 (75 %)	123 (68 %)	113 (49 %)	<0.001
Practice/Implementation				
1. Are nine patient safety solution implemented in my institution?	298 (85 %)	153 (85 %)	166 (72 %)	<0.001
a. Patient identification	250 (71 %)	125 (69 %)	130 (56 %)	<0.001
b. Look-alike sound-alike medication names (LASA)?	107 (30 %)	52 (29 %)	60 (26 %)	0.481
c. Improved hand hygiene to prevent health-care associated infections (HCAI)?	274 (78 %)	137 (76 %)	112 (48 %)	<0.001
d. Performance of correct procedure at correct body site	191 (54 %)	96 (53 %)	65 (28 %)	<0.001
e. Avoiding catheter and tubing mis-connections	106 (30 %)	65 (36 %)	30 (30 %)	<0.001
f. Control of concentrated electrolyte solutions	72 (21 %)	49 (27 %)	25 (11 %)	<0.001
g. Communication during patient hand-overs	206 (59 %)	94 (52 %)	89 (38 %)	<0.001
h. Assuring medication accuracy at transitions in care	158 (45 %)	66 (37 %)	53 (23 %)	<0.001
i. Single use of injection devices	254 (72 %)	123 (68 %)	91 (39 %)	<0.001

*SD* Standard deviation, *IQR* Interquartile range Analyses

^a^Allied health professionals include pharmacists, physical/occupational/speech rapists, biomedical scientists, and dieticians

As shown in Table 1, the first section gathered the participant’s background information such as age, gender and nature of work at the hospital or unit. In the second section, as shown in Table 1, participants were asked about their self-estimated awareness (“Have you heard about patient safety solutions before?”) on the WHO nine patient safety solutions [7]. The participants were required to respond ‘Yes’ or ‘No’. The third section, as shown in Table 1, probed the issues pertinent to implementation or practice (“Are nine patient safety solutions implemented in my institution?) This required them to answer either ‘Yes’ or ‘No’.

The initial draft of the instrument was subjected to a content validity index. Expert agreement was sought from 10 academics that were well-versed in patient’s safety culture. These experts were required to endorse whether the included items were ‘relevant’ or ‘irrelevant’. The agreement of 8/10 experts was set as a benchmark to achieve content validity beyond the 0.05 level of significance as per protocol [11]. Analysis reported the aggregated endorsement of the experts achieved a 0.90 content validity index at 0.05 level of significance. The final questionnaire is shown in Table 1 with 9 items for awareness and 9 items for practice or implementation.

### Participants, recruitment and random selection

The sample size was calculated based on the assumption that the level of knowledge, attitude and perception of the nine patient safety solutions would be around 50 %. In order to estimate this with the precision of 5 and 95 % confidence interval, the study needed to recruit nearly 400 healthcare workers. As the survey was cluster based (hospitals and health centers were considered as clusters) and a design effect of 2 assumed, the sample size required was ammended upwards to 800. The total number of healthcare institutions (primary, secondary and tertiary) under the supervision of the Ministry of Health, was 211 primary healthcare centers and 14 hospitals. Using a random sampling method, one health center was selected from each Wilayat (i.e. town / city) amounting to 59 health centers, while all 14 hospitals were included in the survey. The total number of 800 participants (590 from health centers and 210 from hospitals). The target population for this survey were physicians, nurses and allied healthcare professionals working in the aforementioned facilities. The process of selecting staff from each category was as follows: (i) a minimum of 10 participants from health centers and local hospitals (4 = nurses; 3 = physicians; 3 = allied health care professionals: pharmacist, laboratory technician and radiographer, dietitian, health educator); (ii) a minimum of 15 participants from regional hospitals (5 nurses, 5 physicians, 5 allied health care; (iii) professionals: pharmacist, laboratory technician and radiographer, dietitian, health educator). Selection of participants from hospitals was done randomly from major departments (e.g. 1 physician from internal medicine, 1 from surgery, 1 from child health, 1 from emergency room and 1 from obstetrics and gynecology). Nurses were also selected randomly from varied wards or those serving in outpatient clinics with non-hospitalized patients. The same protocol was applied for allied health care professionals. If their awareness or practical level was lower than 50 % they were analysed in this study.

#### Data collection

The consenting participants were approached in their respective units by the designated researcher in charge of the questionnaire. The researcher explicitly informed the consenting participants that the study was anonymous and voluntary. The participants were informed that they were free to withdraw from the study at any time, without prejudice. The information sought would be aggregated in order to conceal their identity and other personal details. The participants were asked not to discuss the questions amongst themselves in order to avoid peer influence.

### Statistical analysis

To analyse the data, descriptive statistics were used. Frequencies and percentages were reported to illuminate categorical variables. Differences between groups were analyzed using Pearson’s chi-squared tests (or Fisher’s exact tests for cells less than 5). Continuous variables, mean and standard deviation (SD) or median and interquartile range (IQR) were used to summarize the data, as appropriate. Differences between groups were analyzed using uni-variate ordinary least squares (OLS) regression or the Kruskal Wallis test, wherever appropriate. Summative scores for knowledge, attitude and practice were computed. The reliability coefficients between respondents (physicians, nurses and allied health professionals) were calculated using a two analyses variance model, based on 20 subjects in each category from the pilot study data. Cronbach’s alpha, a coefficient of internal consistency, was used to estimate the reliability of the respondents on knowledge, attitude and practice questions. The design effect (intra class correlation (ICC) coefficient) within the health facility was adjusted using hierarchical modeling. The need for random intercept (facility as a cluster) was decided based on the difference between the naïve and random intercept models likelihood ratios. Hierarchical multivariable regression analyses were done using these scores (awareness, attitude and practice) as the dependent variable and age (years) of the participants, type of hospitals (1 = hospital; 2 = health centers), years of experience and type of personnel (1 = nurse; 2 = physicians and 3 = allied health professionals) as explanatory variables. An a priori significance level was set at 0.05. Statistical analyses were performed using Stata version 13.1 (StataCorp, College Station, TX, USA). MLWin software was used to perform hierarchical modeling.

## Results

A total number of 763 staff were recruited giving a response rate of 95 % (763/800). Inter-rater agreement among respondents on the awareness and practice questions was substantial (rho = 0. 93; *p* < 0.001). The majority (46 %) of the participants were nurses with an overall mean age of 33 ± 8 years. Female staff represented 66 % (*n* = 500). Overall, 85 % of the participants stated that they werw familiar with WHO’s nine patient safety solutions aide memoires. Table 1 details the participant’s self-estimated awareness of the nine patient safety solutions.

When each individual item of the nine patient safety solutions was examined the questions regarding participants’ self-estimated awareness (e.g. “*Look-alike sound-alike medication names*” and “*Avoiding catheter and tubing mis-connections*”) were below 50 % threshold operationalized for this study. Items related to the operationalized concept of ‘practice” or implementation (e.g. *“Look-alike sound-alike medication names*?”, “*Avoiding catheter and tubing mis-connections*”, “*Control of concentrated electrolyte solutions*” and “*Assuring medication accuracy at transitions in care*”) were below 50 %. Overall, it was the allied health professionals that exhibited lower awareness and poor practice.

The reliability of responses (ICC) for the knowledge of physicians was 0.82, as in 0.90 and 0.85 for the nurses and allied health professionals respectively, which was statistically significant. The ICC for the attitude domain was 0.53, 0.76, and 0.80 for physicians, nurses and allied health professionals respectively (*p* < 0.001). Similarly, the ICC for the practice domain was 0.87, 0.91, 0.86 for physicians, nurses and allied health professionals respectively (*p* < 0.001).

The mean (sd) of knowledge score was 6.4 (2.9), 5.9 (3.3) and 4.4 (3.1) for nurses, physicians and allied health professionals respectively. Allied health professionals had significantly lower knowledge scores as compared to physicians and nurses (*p* < 0.001). The mean (sd) of the attitude score was 2.7 (1.6); 2.0 (1.2) and 2.6 (1.5) for nurses, physicians and allied health professionals respectively. Thus, physicians had significantly lower attitude score as compared to nurses and health professionals (*p* < 0.001). The mean (sd) of practice score was 12.0 (6.0), 11.2 (5.9) and 7.7 (5.3) for nurses, physicians and health professionals respectively. Both nurses and physicians had a significantly higher score as compared to allied health professionals (*p* < 0.001).

The results of multivariable regression analyses are presented in Table 2. The hierarchical multivariable analyses for awareness suggested that as the professional's age increased, the awareness score also increased significantly (*p* < 0.001). Primary Health Care center’s staff had significantly higher knowledge than the hospital staff (*p* < 0. 001). The allied health professionals had significantly lower awareness than the physicians and nurses (*p* < 0. 001). The regression analyses for attitude suggested that as age increased the attitude decreased significantly (*p* < 0.001) and there was no significant difference between hospitals and Primary Health Care centers, after adjusting for type of workers, years of experience and age of the subjects.

**Table 2:** Regression analyses for awareness and practice on patient safety among different subtypes of healthcare workers in Oman (*N* = 763)

Predictors	Regression^c^ coefficient	SE^c^	*P* value
Knowledge Score:			
Age (years)	.064	.015	<0.001
Type of workers^a^	-.956	.125	<0.001
Numbers of years at this hospital	.035	.034	0.401
Numbers of years at this hospital in the present specialization/unit	-.023	.039	0.691
Type of Health Facility^b^	.915	.416	<0.001
Awareness Score:			
Age (years)	-.041	.008	<0.001
Type of workers	-.099	.065	0.101
Numbers of years at this hospital	.016	.017	0.479
Numbers of years at this hospital in the present specialization/unit	.036	.020	0.066
Type of Health Facility^b^	-.044	.215	0.472
Practice Score:			
Age (years)	.157	.030	<0.001
Type of workers	-2.12	.24	<0.001
Numbers of years at this hospital	-.002	.065	0.904
Numbers of years at this hospital in the present specialization/unit	-.034	.075	0.723
Type of Health Facility ^b^	1.26	.738	0.09

^a^ Type of workers: 1 = Nurse; 2 = Physicians; 3 = Others

^b^ Type of Health facility: 1 = Hospitals; 2 = Health centres

^c^ Regression coefficients and SE based on Hierarchical modelling

The regression analyses for practice suggested that as the age increased, practical knowledge increased significantly (*p* < 0.001). Physicians and nurses had significantly higher practical knowledge as compared to allied health professionals (*p* < 0.001). However, there was no difference in practical knowledge reported between physicians and nurses. Participants working in the Primary Health Care centers had significantly higher practical knowledge as compared to hospitals (*p* < 0.01).

## Discussion

Presently, within Arab countries, this study serves as a pioneer in assessing healthcare workers’ self-estimated awareness and practice of nine patient safety solutions. Globally there is a paucity of studies on the nine patient safety solutions except for some studies that have examined some of the components or ‘derivatives’ of the lifesaving patient solutions [12, 13]. Previously, the bulk of the research, including some from Oman, has focused on ‘patient safety culture’ [14, 15] which is limited to an organization’s culture towards safety measures.

In our present study, the participants (physicians, nurses and allied health professionals) are representative of the healthcare workforce in Oman. Nurses and physicians constitute 28 % of the Ministry of Health workforce respectively[16], and hence the representation in the present study. It is worthwhile to note that this present study has a significant number of young adult and female participants. This also reflects the demographics of the workforce in the country [17]. It remains to be seen whether the ‘younger generation’ does remain abreast with the nine patient safety solutions aide memoires stipulated by World Health Organization [2].

In addition to comparing the self-estimated awareness of the nine patient safety solutions among nurses, physicians and allied health professionals, it would be interesting to consider other socio-demographic factors. The effect of age on self-estimated awareness and practice is robustly in this study; age could be considered as a confounder. Therefore, this limits the generalization of the study since the cohorts were not homogenous regarding age grouping. Despite this caveat, implications of the age factor are worthwhile to speculate upon. It can be suggested that elder employees reflected greater experience in the workplace, which has a positive effect on their self-estimated awareness and practice of the nine life-saving patient safety solutions. This is seemingly consistent with the common view that the longer the experience, the more likely it is to shape someone’s awareness and practice. However, although self-estimated awareness and practice have a positive impact on the implementation of the nine patient safety solutions, awareness appears not to be influenced by age. This study indicates that awareness of the nine patient safety solutions decreases with age. To derive a correlation between age and attitude, it is necessary to refer to social science studies; one such study has clearly indicated that younger age groups are amenable to attitude change, whereas the older population’s attitude tends to be more stable or firmly consolidated and therefore not amenable to change [18].

Another phenomenon that has emerged from this study is the preponderance of staff in the Primary Healthcare centers who have higher self-estimated awareness with regard to patient safety issues. In this study PHCs were more often staffed by healthcare professionals who are older than the staff in hospital. This could be indicative of the greater awareness in PHC’s. It is also possible that PHC’s might have fewer patients compared to hospitals, which in turn could allow the health personnel in Primary Health Care centers to be more vigilant for life-saving patient safety solutions.

Limitations inherent in this type of psychosocial study need to be highlighted. The first issue is regarding social desirability. There is a risk that some respondents may feel that giving an ‘honest’ view would render incompetent or that their unit/department/healthcare-center would be viewed as having suboptimal patient safety measures. It is common in surveys for participants to self-estimate their awareness and attitude in a manner that will be viewed favorably by others. Thus, respondents may over-estimate their “good behavior” or, conversely under-estimate their “bad behavior”. Thus, as is common in psychosocial studies, this study may also be marred by social desirability bias [19]. The assumption is that it may be easier to be honest, if you are young and newly employed compared to someone who has been in the organization for a long time. In addition to social desirability bias, there is no indication that an individual'sattitude translates into behavior [20] and thus, for the present context, there may be a disjunction between awareness and practice. Hence, generalization of this study should be reviewed within the context of limitations inherent in a study, suggestive of social desirability bias and the fact that attitude does not always translate into practice.

## Conclusion

Despite the above-mentioned caveats, this is the first study to examine the self-estimated awareness and practice among different groups of healthcare professionals in Oman. The Primary Health Centre staff demonstrated higher self-estimated awareness compared to Hospital Staff. Variables such as age, working in a hospital or primary health centers appears to have direct bearing on participant’s self-estimated awareness and practice. This study lays the groundwork for further scrutiny on World Health Organization’s nine patient safety solutions. This study suggests that concerted efforts should be made to heighten patient safety in Oman.

## Additional file

Additional file 1: Awareness and practice of nine World Health Organization’s patient safety solutions. (DOCX 34 kb)

## References

[1] Balkhair A, Al-Farsi YM, Al-Muharrmi Z, Al-Rashdi R, Al-Jabri M, Neilson F, Al-Adawi SS, El-Beeli M, Al-Adawi S (2014). Epidemiology of multi-drug resistant organisms in a teaching hospital in Oman: a one-year hospital-based study. ScientificWorldJournal.

[2] O’Leary DS (2008). Patient safety: the search for global solutions. World Hosp Health Serv.

[3] Donaldson LJ, Fletcher MG (2006). The WHO World Alliance for Patient Safety: towards the years of living less dangerously. Med J Aust.

[4] Pittet D, Donaldson L (2006). Challenging the world: patient safety and health care-associated infection. Int J Qual Health Care.

[5] Arias CA, Murray BE (2009). Antibiotic-resistant bugs in the 21st century--a clinical super-challenge. N Engl J Med.

[6] Al-Muharrmi Z, Rafay A, Balkhair A, Jabri AA (2008). Antibiotic combination as empirical therapy for extended spectrum Beta-lactamase. Oman Med J.

[7] WHO Collaborating Center for Patient Safety’s nine life-saving Patient Safety Solutions. Joint Commission journal on quality and patient safety / Joint Commission Resources, 2007; 33 (7): 427–462. 10.1016/s1553-7250(07)33126-7 17712914

[8] World Health Organization. The nine Patient Safety Solutions, 2007. Accessed from http://www.who.int/patientsafety/events/07/02_05_2007/en/ on 23 June 2016.

[9] Stocks SJ, Giles SJ, Cheraghi-Sohi S, Campbell SM (2015). Application of a tool for the evaluation of public and patient involvement in research. BMJ Open.

[10] Kinninmont J (2015). Chatham House Report: Future Trends in the Gulf.

[11] Lynn MR (1986). Determination and quantification of content validity. Nurs Res.

[12] Calligaris L, Quattrin R, Londero C, Farneti F, Panariti M, Brusaferro S (2007). Clinical risk management and nutritional therapy in the near future: What are the prospects?. Nutr Ther Metab.

[13] Mackenzie CF, Shander A (2008). What to do if no blood is available but the patient is bleeding?. South Afr J Anaesth Analg.

[14] Ammouri AA, Tailakh AK, Muliira JK, Geethakrishnan R, Al Kindi SN (2015). Patient safety culture among nurses. Int Nurs Rev.

[15] Al-Mandhari A, Al-Zakwani I, Al-Kindi M, Tawilah J, Dorvlo AS, Al-Adawi S (2014). Patient safety culture assessment in Oman. Oman Med J.

[16] Annual Health Report 2013. Ministry of Health, Sultanate of Oman. Accessed from https://www.moh.gov.om/en/web/directorate-general-of-planning/resources. 16 Apr 2015

[17] Alshishtawy MM (2010). Four Decades of Progress: Evolution of the health system in Oman. Sultan Qaboos Univ Med J.

[18] Tyler TR, Schuller RA (1991). Aging and Attitude Change. J Pers Soc Psychol.

[19] Krumpal I (2013). Determinants of social desirability bias in sensitive surveys: a literature review. Qual Quant.

[20] Ajzan I, Fishbein M (1980). Understanding attitudes and predicting social behavior.

