# Supplementary material for: Awareness and implementation of nine World Health Organization’s patient safety solutions among three groups of healthcare workers in Oman
Source: BMC Health Serv Res. 2016 Sep 30;16:533. doi: 10.1186/s12913-016-1771-1 (PMC5045576; doi:10.1186/s12913-016-1771-1)
Supplement: Additional file 1: — Awareness and practice of nine World Health Organization’s patient safety solutions. (DOCX 34 kb) [file 12913_2016_1771_MOESM1_ESM.docx]

**Awareness and practice of nine World Health Organization’s patient safety solutions**

**Background Information**

Age (years)

Gender: a. Male b. Female

Scope of work at your hospital: (Please select ONE answer that best describes your staff position):

| a | Nurse |  |
| --- | --- | --- |
| b | Physician |  |
| c | Intern (doctor) |  |
| d | Physical, Occupational or Speech therapist |  |
| e | Biomedical Scientist |  |
| f | Pharmacist |  |
| g | Dietician |  |
| h | Other, Please specify |  |

| How long have you worked in this institution? | Years |
| --- | --- |
| How long have you worked in your current institution work area/unit? | Years |

**Awareness**

| Are you aware or have you heard of nine patient  safety solutions before |  | Yes  No |
| --- | --- | --- |

If **YES**, please select a solution/solutions that you are aware of
(you may select more than one solution)

|  | Patient identification |
| --- | --- |
|  | Look alike |
|  | Improved hand hygiene to prevent health care Associated infections |
|  | Performance of Correct Procedure at Correct Body Site |
|  | Avoiding Catheter and Tubing Mis-Connections |
|  | Control of Concentrated Electrolyte Solutions |
|  | Communication during Patient Handovers |
|  | Assuring Medication Accuracy at Transitions in Care |
|  | Single use of injection devices |

**Practice and Implement**

| Have nine patient safety solutions been implemented in your institution?    Yes  No |
| --- |

If **YES**, please select a solution/solutions that are practiced or implemented in your practice
(you may select more than one solution)

|  | Patient identification |
| --- | --- |
|  | Look Alike, Sound Alike medication names (LASA) |
|  | Improved Hand Hygiene to Prevent Health Care-Associated Infections |
|  | Performance of Correct Procedure at Correct Body Site |
|  | Avoiding Catheter and Tubing Mis-Connections |
|  | Control of Concentrated Electrolyte Solutions |
|  | Communication during Patient Handovers |
|  | Assuring Medication Accuracy at Transitions in Care |
|  | Single use of injection devices |
